# Strangulated Small Bowel Obstruction Involving the External Iliac Artery after Pelvic Lymph Node Dissection: A Case Report on Stepwise Intraoperative Decision-Making to Prevent Vascular Injury

**DOI:** 10.70352/scrj.cr.26-0300

**Published:** 2026-06-23

**Authors:** Tomoyasu Kashiwagi, Yoshiaki Fujimoto, Yuya Ono, Takuma Izumi, Yukihiko Aoyagi, Fumihiko Hirai, Rintaro Yoshida, Takuya Honboh, Noboru Harada, Hiroyuki Itoh, Noriaki Sadanaga, Tomoharu Yoshizumi

**Affiliations:** 1 Department of Surgery, Saiseikai Fukuoka General Hospital, Fukuoka, Fukuoka, Japan; 2 Department of Surgery and Science, Graduate School of Medical Sciences, Kyushu University, Fukuoka, Fukuoka, Japan

**Keywords:** internal hernia, external iliac artery, pelvic lymph node dissection, small bowel obstruction, vascular injury, surgical strategy

## Abstract

**INTRODUCTION:**

Postoperative anatomical-space–related internal herniation/strangulation after pelvic lymph node dissection (PLND) is uncommon but may be associated with a risk of inadvertent injury to major vessels or urinary tract-related structures.

**CASE PRESENTATION:**

A 77-year-old man with a history of robot-assisted radical cystectomy with ileal conduit diversion and PLND presented with severe abdominal pain. Contrast-enhanced CT revealed a closed-loop small bowel obstruction (SBO) with suspected strangulation adjacent to the right external iliac artery. Emergency laparotomy revealed extensive ischemic and necrotic changes of the small bowel in the right pelvis. Gentle reduction was first attempted but was unsuccessful. Because the constricting structure could not be adequately identified and was considered to potentially involve critical structures requiring preservation, premature direct release or forceful division was avoided. Because direct visualization of the dorsal pelvic structures was limited, CT images were re-reviewed intraoperatively to confirm that the urinary diversion-related structures were separate from the clearly nonviable target bowel. Initial intraloop bowel transection was then performed within the nonviable segment to reduce tension and improve visualization, allowing subsequent identification of the right external iliac artery as the constricting structure. Approximately 230 cm of small bowel was resected based on bowel viability, while more than 200 cm of small bowel was preserved. The artery was preserved and fixed to the pelvic wall. The postoperative course was uneventful.

**CONCLUSIONS:**

This case highlights a postoperative anatomical-space–related internal herniation/strangulation after PLND. When strangulated SBO occurs adjacent to a major vessel or other critical structures, premature direct release of an inadequately identified constricting structure should be avoided. In selected cases in which gentle reduction is impossible, initial intraloop bowel transection may be considered as a limited bail-out option only after confirming that the clearly nonviable target bowel is anatomically separate from urinary diversion-related structures. This approach may reduce tension and improve visualization for subsequent identification of the constricting structure.

## Abbreviations

BUN
: blood urea nitrogen
Cr
: creatinine
CRP
: C-reactive protein
PLND
: pelvic lymph node dissection
SBO
: small bowel obstruction
WBC
: white blood cell count

## INTRODUCTION

Internal hernia is a recognized cause of SBO and may rapidly progress to strangulation and bowel necrosis.^1,2)^ In a postoperative setting, surgically created defects or abnormal intra-abdominal spaces may contribute to internal herniation or volvulus after abdominal surgery.^3)^ PLND can alter pelvic anatomy by exposing major vessels and creating potential spaces around the pelvic sidewall. Although several cases of internal hernia associated with skeletonized iliac vessels after pelvic lymphadenectomy have been reported, strangulated SBO involving the external iliac artery remains uncommon and poses a risk of inadvertent vascular injury.^4–7)^ Herein, we report a case of postoperative anatomical-space-related internal herniation/strangulation after PLND, focusing on intraoperative decision-making to avoid vascular and urinary tract injury when the constricting structure cannot be safely identified.

## CASE PRESENTATION

A 77-year-old man presented with acute-onset severe upper abdominal pain. He had undergone robot-assisted radical cystectomy with ileal conduit diversion and PLND 174 days before presentation, resulting in surgically altered pelvic anatomy.

On admission, he had persistent severe abdominal pain despite the administration of analgesics. His vital signs were as follows: body temperature, 35.7°C; heart rate, 67 beats/min; blood pressure, 162/92 mmHg; and oxygen saturation, 98% on room air. Laboratory findings revealed leukocytosis (WBC, 12200/μL), elevated BUN (49.5 mg/dL), elevated Cr (2.00 mg/dL), and an increased serum lactate level (4.4 mmol/L), whereas CRP was not markedly elevated (0.17 mg/dL). These findings raised concern for intestinal ischemia.

Contrast-enhanced CT revealed a closed-loop configuration of the small bowel with decreased bowel wall enhancement, raising suspicion of strangulated SBO (**Fig. 1A**–**1C**). The affected bowel segment was located in the right pelvis, and the mesentery appeared to be interposed between the right external iliac artery and the lateral pelvic wall. The preoperative differential diagnoses included adhesive SBO, strangulated SBO, internal herniation, and incarceration of the small bowel or mesentery into a postoperative pelvic space. Although vascular involvement was considered based on the anatomical relationship on CT, it was difficult to definitively identify the external iliac artery as the constricting structure preoperatively. However, the ileal conduit and the ureteroileal region were visualized separately from the strangulated small bowel segment on CT (**Fig. 1D** and **1E**), supporting the preoperative anatomical assessment that the urinary diversion-related structures were not included in the closed-loop segment.

**Fig. 1:**
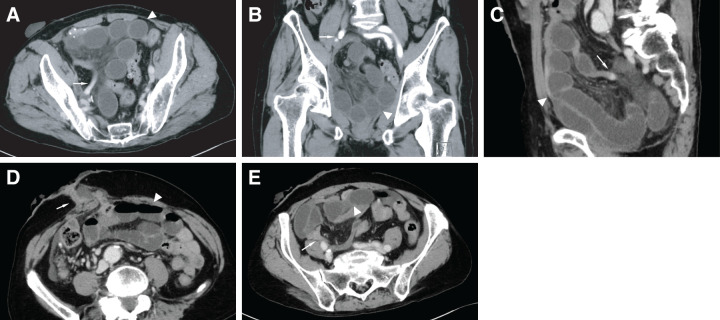
Contrast-enhanced CT findings. (**A**–**C**) Axial, coronal, and sagittal views. The arrow indicates the right external iliac artery, and the arrowheads indicate the small bowel with decreased enhancement, forming a closed-loop configuration. The bowel appeared to be entrapped between the artery and the pelvic wall, suggesting postoperative anatomical-space–related internal herniation/strangulation. (**D**) Axial view showing the ileal conduit (arrow) separately from the strangulated small bowel segment (arrowhead). (**E**) Axial view showing the ureteroileal region (arrow) separately from the strangulated small bowel segment (arrowhead). These findings supported the anatomical assessment that the urinary diversion-related structures were separate from the strangulated bowel segment.

An emergency laparotomy was performed. Upon entering the abdominal cavity, a large amount of hemorrhagic ascites and a markedly congested and distended small bowel loop were observed, occupying the operative field and limiting visualization (**Fig. 2A**). The incarcerated bowel appeared to be constricted in the right pelvis; however, the constricting structure could not be clearly identified at this stage.

**Fig. 2:**
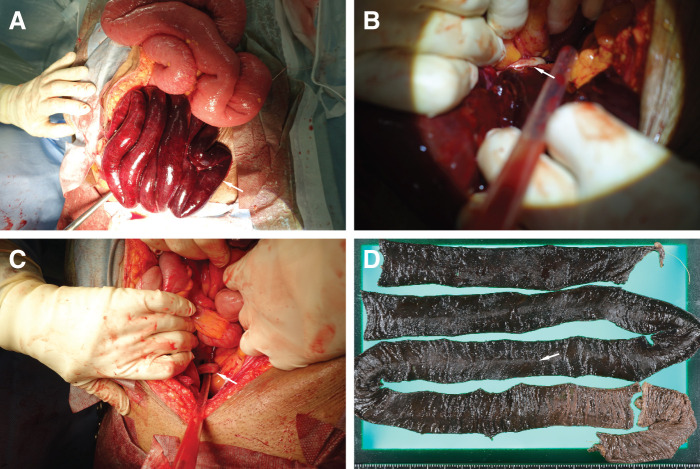
Intraoperative findings and resected specimen of strangulated SBO involving the external iliac artery. (**A**) Intraoperative view showing markedly congested closed-loop small bowel. The arrow indicates the congested segment, and the distended bowel occupied the operative field, limiting visualization. (**B**) Intraoperative identification of the hernia orifice. The arrow indicates the right external iliac artery forming the hernia orifice. The small bowel was herniated beneath the artery, resulting in strangulation within the postoperative pelvic space. (**C**) Intraoperative findings after bowel resection. The hernia orifice was clearly identified dorsal to the right external iliac artery (arrow), confirming that the artery formed the hernia orifice. (**D**) Resected specimen showing approximately 230 cm of small bowel with extensive ischemic and necrotic changes. The arrow indicates the initial intraloop transection site, which was limited to the clearly identifiable nonviable bowel segment without transection of the deep pelvic constricting structure. After release of the strangulation, additional proximal and distal bowel segments were resected during reconstruction to establish definitive viable transection margins. The final oral transection line was approximately 200 cm distal to the ligament of Treitz, and the final anal transection line was approximately 60 cm proximal to the ileocecal valve. SBO, small bowel obstruction

Gentle traction was first attempted to reduce the incarcerated bowel from the constricting structure; however, safe reduction could not be achieved. Because the constricting structure could not be adequately identified and was considered to potentially involve critical structures requiring preservation, premature direct release or forceful division was avoided. Because direct visualization of the dorsal pelvic structures was limited by the distended and ischemic bowel, the CT images were re-reviewed intraoperatively to confirm the anatomical separation of the clearly nonviable target bowel from the ileal conduit and other urinary diversion-related structures. On this basis, initial intraloop bowel transection was performed only within the clearly identifiable nonviable small bowel segment to reduce tension and improve visualization, without transection of the deep pelvic constricting structure or manipulation around the ileal conduit or the ureteroileal region (**Figs. 2D** and **3**). Ureteral catheterization or stent placement was not performed because the urgent clinical setting required prompt surgical intervention and the anatomical separation of these structures from the target bowel had been confirmed by CT assessment before and during surgery.

**Fig. 3:**
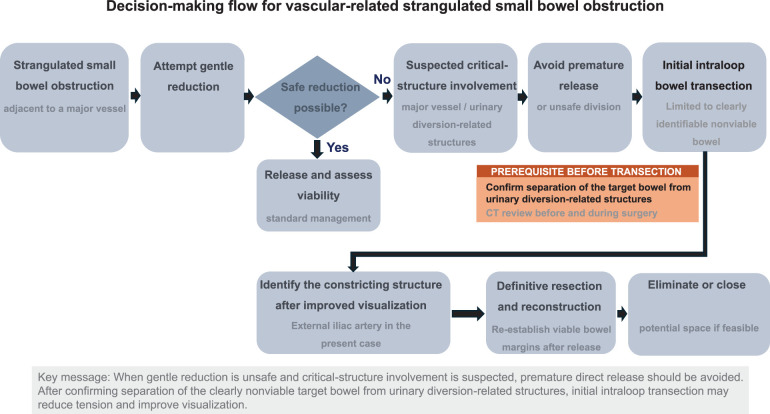
Decision-making flow for vascular-related strangulated SBO. Gentle reduction should first be attempted when strangulated SBO occurs adjacent to a major vessel. If safe reduction is not possible and involvement of critical structures, including major vessels or urinary diversion-related structures, is suspected, premature direct release or unsafe division should be avoided. In patients with urinary diversion, separation of the clearly nonviable target bowel from urinary diversion-related structures should be confirmed by appropriate preoperative and intraoperative image review before transection. Initial intraloop bowel transection limited to the clearly identifiable nonviable bowel may reduce tension and improve visualization, allowing subsequent identification of the constricting structure. Definitive viable bowel margins should be established during reconstruction after release of the strangulation, followed by closure or elimination of the potential space when feasible. SBO, small bowel obstruction

After bowel transection, the tension on the incarcerated bowel was reduced, and visualization of the right pelvic space improved. This allowed safe identification of the right external iliac artery as the constricting structure forming the hernial orifice (**Fig. 2B**). Further inspection revealed that the hernial orifice was located dorsal to the artery, confirming the anatomical relationship between the external iliac artery, the pelvic wall, and the incarcerated bowel (**Fig. 2C**). After release of the strangulation, additional proximal and distal bowel resection was performed during reconstruction to establish definitive viable bowel margins.

In total, approximately 230 cm of small bowel with extensive ischemic and necrotic changes was resected based on the assessment of bowel viability (**Fig. 2D**). The resected specimen included the initially transected nonviable intraloop segment and the additionally resected proximal and distal segments. The final oral transection line was approximately 200 cm distal to the ligament of Treitz, and the final anal transection line was approximately 60 cm proximal to the ileocecal valve; therefore, more than 200 cm of small bowel was preserved, and the risk of short bowel syndrome was considered low. The right external iliac artery was preserved. To prevent recurrence, the adventitia of the artery was fixed to the pelvic wall using 5-0 absorbable sutures without excessive tension. Intestinal continuity was restored using a functional end-to-end anastomosis.

The postoperative course was uneventful. Oral intake was resumed on POD 3, and the patient was discharged on POD 10 without complications. No early clinical signs suggestive of short bowel syndrome were observed during the postoperative hospital stay.

## DISCUSSION

This case represents a postoperative anatomical-space–related internal herniation/strangulation rather than a conventional internal hernia through a mesenteric or peritoneal defect. In the present case, the small bowel and mesentery became incarcerated in a potential space between the right external iliac artery and the pelvic wall after PLND. Although internal hernia is a recognized cause of SBO, involvement of a major vascular structure as part of the constricting anatomy is uncommon and may carry a substantial risk of intraoperative vascular injury.^1,2)^

PLND can alter retroperitoneal anatomy by removing perivascular connective tissue and exposing major vessels, thereby creating potential spaces around vascular structures. In a broader postoperative context, surgically created mesenteric defects or abnormal intra-abdominal spaces have been reported as causes of internal hernia or volvulus after abdominal surgery.^3)^ In the present case, the mechanism was distinct from a typical mesenteric-defect-related internal hernia, but it similarly involved incarceration of the small bowel and mesentery into a surgically altered postoperative space.

Several case reports have described internal hernias associated with skeletonized iliac vessels after pelvic lymphadenectomy, including those beneath or behind the external iliac artery.^4–7)^ These reports suggest that, when surrounding supporting tissue is removed, exposed vascular structures may become part of a hernia orifice or constricting anatomy. In the present case, preoperative CT suggested closed-loop obstruction adjacent to the right external iliac artery, but definitive identification of the artery as the constricting structure was difficult before surgery. This diagnostic uncertainty represents an important intraoperative pitfall because premature direct release or division of an inadequately identified constricting structure may result in vascular or urinary tract injury.

In the present case, a resection-first strategy was not selected as the initial default approach. Gentle reduction of the incarcerated bowel was first attempted but was unsuccessful. Because the constricting structure could not be adequately identified and was considered to potentially involve critical structures requiring preservation, premature direct release or forceful division was avoided. As direct visualization of the dorsal pelvic structures was limited, the preoperative CT images were re-reviewed intraoperatively to confirm that the clearly nonviable target bowel was anatomically separate from the urinary diversion-related structures before transection. Initial intraloop bowel transection, limited to the clearly identifiable nonviable small bowel segment, reduced tension and improved visualization, thereby allowing subsequent identification of the right external iliac artery as the constricting structure. As illustrated in **Fig. 3**, this stepwise approach may be considered a limited bail-out option in selected cases when safe reduction is impossible and critical-structure involvement is suspected.

Importantly, bowel transection first should not be interpreted as permitting transection under uncertain anatomy. In patients with urinary diversion, anatomical separation of the clearly nonviable target bowel from urinary diversion-related structures must be assessed before transection. In the present case, CT assessment before surgery and intraoperative re-review of the CT images showed that the ileal conduit and ureteroileal region were separate from the closed-loop ischemic small bowel. The initial transection was limited to the identifiable nonviable intraloop bowel segment and did not involve transection of the deep pelvic constricting structure or manipulation around the ileal conduit or ureteroileal region. No prophylactic ureteral catheter or stent was used because emergency surgery was required and the anatomical separation relevant to the planned limited transection had been confirmed on CT before and during surgery. Thus, bowel transection first should be regarded as a tension-reducing step after identification of the target ischemic bowel segment and confirmation of its separation from urinary diversion-related structures, rather than as a substitute for careful anatomical assessment of the urinary diversion and its vascular supply.

Although approximately 230 cm of small bowel was resected in this case, the extent of resection was determined based on bowel viability rather than on the resection-first strategy itself. The initial intraloop transection was performed as a limited tension-reducing maneuver and did not define the final extent of bowel resection; after release of the strangulation, definitive viable bowel margins were established during reconstruction. Bowel preservation was considered; however, safe reduction was not possible, and the involved bowel showed extensive ischemic and necrotic changes. Because more than 200 cm of small bowel was preserved, the risk of short bowel syndrome was considered low. Thus, the role of bowel transection first should be limited to reducing tension, improving visualization, and enabling safer assessment of bowel viability in selected high-risk situations.

In the era of robotic pelvic surgery, the number of minimally invasive pelvic procedures has been steadily increasing, as demonstrated by recent national registry data.^8)^ In such procedures, PLND may expose iliac vessels and leave potential spaces around the pelvic sidewall. Although this single case cannot establish a causal relationship or define an optimal preventive measure, surgeons should be aware that exposed vessels and residual postoperative spaces may allow small bowel or mesenteric incarceration. When a potential space remains after PLND, elimination or closure of the space, including peritonealization when technically feasible, may be considered as a preventive option.

This case highlights the importance of stepwise intraoperative decision-making when strangulated SBO occurs adjacent to a major vessel or other critical structures. In selected cases in which gentle reduction is impossible and direct release is considered unsafe, initial intraloop bowel transection, after confirmation that the target bowel is separate from urinary diversion-related structures, may help reduce tension and improve visualization, allowing subsequent identification of the constricting structure without premature manipulation of the deep pelvic constricting site. In addition, elimination or closure of the potential space may be considered when feasible to reduce the possibility of recurrent incarceration, although its preventive effect cannot be determined from a single case. The educational value of this case lies not only in its unusual anatomical configuration but also in the decision-making process used to avoid inadvertent vascular or urinary tract injury.

## CONCLUSIONS

This case highlights a postoperative anatomical-space–related internal herniation/strangulation after PLND. When strangulated SBO occurs adjacent to a major vessel or other critical structures, premature direct release of an inadequately identified constricting structure should be avoided. In selected cases in which gentle reduction is impossible, initial intraloop bowel transection may be considered as a limited bail-out option only after confirming that the clearly nonviable target bowel is anatomically separate from urinary diversion-related structures. This approach may reduce tension and improve visualization for subsequent identification of the constricting structure.

## DECLARATIONS

### Availability of data and materials

The datasets supporting the conclusions of this study are included within this article.
